# Promoting Positive Psychology Using Social Networking Sites: A Study of New College Entrants on Facebook

**DOI:** 10.3390/ijerph110504652

**Published:** 2014-04-29

**Authors:** Shu-Man Chang, Yung-Hsiu Lin, Chi-Wei Lin, Her-Kun Chang, Ping Pete Chong

**Affiliations:** 1 Graduate Institute of Business and Management, Chang Gung University, Taoyuan 33302, Taiwan; E-Mail: amanda@mail.cgu.edu.tw; 2 Center for General Education, Chang Gung University, Taoyuan 33302, Taiwan; 3 Department of Information Management, Ta-Hwa University of Technology, Hsinchu 30740, Taiwan; E-Mail: lin021060@gmail.com; 4 Department of Counseling and Clinical Psychology, National Dong-Hwa University, Hualien 97401, Taiwan; E-Mail: chiwei@mail.ndhu.edu.tw; 5 Department of Information Management, Chang Gung University, Taoyuan 33302, Taiwan; 6 College of Management and Design, Ming Chi University of Technology, New Taipei 24301, Taiwan; E-Mail: chong@mail.mcut.edu.tw

**Keywords:** positive psychology, online social network, Facebook, new college entrant, engagement, social influence

## Abstract

This study explores the potential of promoting college students’ positive psychological development using popular online social networks. Online social networks have dramatically changed the ways college students manage their social relationships. Social network activities, such as checking Facebook posts dominates students’ Internet time and has the potential to assist students’ positive development. Positive psychology is a scientific study of how ordinary individuals can apply their strength effectively when facing objective difficulties and how this capability can be cultivated with certain approaches. A positive message delivery approach was designed for a group of new college entrants. A series of positive messages was edited by university counselors and delivered by students to their Facebook social groups. Responses from each posted positive messages were collected and analyzed by researchers. The responses indicated that: (1) relationships of individual engagement and social influence in this study can partially explain the observed student behavior; (2) using class-based social groups can promote a positive atmosphere to enhance strong-tie relationships in both the physical and virtual environments, and (3) promoting student’s positive attitudes can substantially impact adolescents’ future developments, and many positive attitudes can be cultivated by emotional events and social influence.

## 1. Introduction

Positive psychology is the scientific study of how ordinary individuals apply their strength and virtues effectively when facing objective difficulties and how this subjective capability can be properly cultivated with certain approaches [1]. The capability enables individuals to adapt themselves to different life situations, feel satisfied with their experiences, and not trapped by depressive conditions [2]. Psychological practitioners have typically devoted their attention to diagnosing and treating human pathological errors rather than understanding how normal individuals develop positive capabilities to cope with possible mental illnesses [3]. The development of the latter is closely related to an individual’s emotions, his/her traits, and is subtly embedded in social contexts [4]. As online social network services (SNS), such as Facebook, extensively penetrate into individuals’ daily activities today, practitioners and researchers of positive psychology may have the opportunity to design proper initiatives for positive development by means of observing the relationship between individual behavior and social influence [5,6].

Positive psychology studies how normal people flourish under certain conditions [7], and it provides a robust theoretical framework for practitioners in various domains, such as learning [8], coaching [9] and healthcare [10]. This framework focuses on three areas of human experience. The first area is positive emotions at the subjective level, such as happiness, love, and contentment with lives that enables individuals to value subjectively and feel satisfied on their past experiences, happy about current situations, and optimistic about the future [2]. The second is positive traits at an individual level, such as courage, persistence, and honesty that are good virtues and values helping individuals to conceptualize good lives [1]. The last is positive institutions at a group level, such as civic virtues, healthy work environments, and positive communities that provide individuals with a benign social context to have positive social influences [7]. The framework can be used as a guideline for practitioners and researchers in defining the scope of their interests and for cultivating certain approaches for positive psychology development.

The three areas identified above facilitate a systemic framework for individuals to use their positive traits to pursue positive emotions within the context of positive institutions and to realize their subjective happiness and well-being [9]. This framework has been enriched over time; for examples, a catalogue of core virtues and character defined in positive strengths as “Values in Action” was published [11], and there are studies in the relationship between positive emotion and institution performance [9] as well as the influence of external events toward individual internal emotion [12]. Recently, peer-support programs are emerging as highly effective and empowering ways for people to manage their health related issues and benefit from a supportive social context. The popularity of online social networks can have the potential to create supportive social contexts [13], especially for adolescents in different phases of schooling, by helping their positive development. The activities involved in that social context including their individual engagement and social influence can have considerable impact on the future development of their strength and behavior [14,15].

### 1.1. Social Networks in College

For many adolescents, entering college is a lifestyle transition, leaving high school friends and close parent support behind to move to an unfamiliar and perhaps remote environment. Colleges organize supportive activities; for examples, orientation programs to provide useful information for new entrants about their daily schedules, and psychological consulting offices at universities also may conduct mental health evaluations to screen at-risk students for further assistance [16]. However, programs on motivating students’ positive trait development through informal channels have yet to be fully explored. The challenges for using students’ information channels to distribute messages for positive development are basically issues of engagement and influence [17]. Students can decide freely whether to participate in the events, share with others, or simply ignore them altogether [18]. As Facebook has become one of the most popular informal channels for students [13], college administrations may consider using its potential social influence to assist new students in their life adjustments and positive development [17,19].

Measuring activities on Facebook has attracted many practitioner and researcher attentions [10,20]. In general, Facebook provides functions for its users to post information, maintain friendships, and communicate with others [21]. In Facebook, members of a *Group* can respond to a post on the *Wall* by clicking the Like link to indicate support to a message or simply “have read.” They can also write opinions to a post using the Comment function and share their opinions to specific online friends, groups, or the public [13]. These responses via Like and Comment functions may be used as crucial metrics for measuring the effectiveness of online events [19]. Most online events on Facebook are fast-paced and individuals would soon lose interest if an event does not stimulate discussions among members or show influences in the social groups [17]. In other words, individual engagement with certain events may be influenced by activities in social groups, and the social influence can only be stimulated by a sufficient number of individual engagements [10].

Some researchers have ventured into using SNS as a platform for academic and educational applications. Forkosh-Baruch and Hershkovitz [22] studied the scholarly information sharing within academic communities, and Mazzoni [21] studied how SNS affects the social capital in the transition of emerging adults from high school to university, for examples. However, how social influence from SNS may impact individual behavior—especially the positive psychological development—has yet to be fully explored.

### 1.2. Objectives

This study designed an online positive message delivery event for a group of new college entrants through Facebook social groups to explore how this new platform may be used to provide positive message to enhance the development of positive capabilities. Responses from the new entrants were collected and analyzed. This study will measure individual engagement and estimate social influence from the responses of each message posted. An acceptance evaluation will be conducted to estimate students’ attitudes toward this formal event.

## 2. Experimental Section

### 2.1. Population and Procedure

In February 2012, a group of new college entrants (N = 150) in a northern Taiwan university were invited to participate in this online social network study. The group consists of two classes, one from the medical school and the other from the management school. The researchers presented study details including research objectives and procedures to each class and answered students’ concerns regarding information security and privacy issues. In this study, researchers, class teachers and students’ parents were excluded from the Facebook group in which students congregate so that discussions may be held without restraint. The participation was not mandatory, and written consents were obtained from participants. One class secretary was elected by students in each class separately as a message carrier, bridging the communications between researchers and the class Facebook groups. A survey was conducted before the experiment to collect participants’ demographic data, time spent on Internet each week, and Facebook experiences. This study was approved by the university’s psychological consulting office and was conducted in conjunction with their routine new entrants' mental health tests. The researchers worked closely with the psychologist and counselor at the university counseling center throughout this study.

### 2.2. Study Design

This study was conducted in close collaboration with university staff including university counselors and class teachers on promoting positive development to a group of new entrants with a series of online positive messages. In general, at the beginning of each academic year university counselors would conduct a mental health evaluation to screen new entrants for those who might need mental care [16]. In conjunction with this evaluation, this study aims to advance the understanding on how to assist ordinary new entrants on positive psychological development. A series of positive messages were collected and organized to deliver to the students’ online social network. The messages were delivering on the class groups’ Facebook “*Walls*” by class secretaries. These “*Walls*” were used as a major platform for sharing class- or non-class-related information and students could browse the posted messages individually. Since students were participating in similar activities and classes during their freshman year, they tended to interact with each other frequently. The interactions could develop strong-tie online relationships with their classmates and may stimulate social influence among group members. 

**Figure 1:**
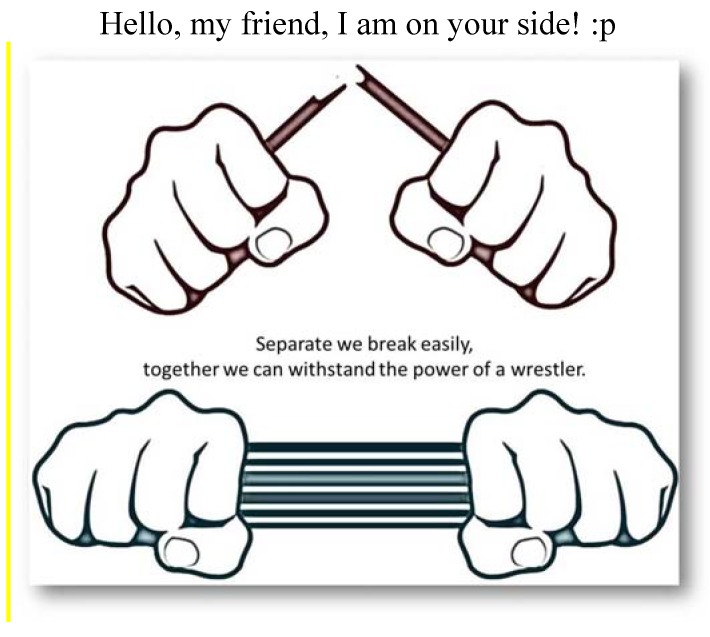
An illustration of positive messages.

Thirty eight positive messages ranging from direct aphorisms or mottos to soft reminders or prose were collected from non-profit organizations and Internet resources in relation to promoting positive developments on adolescents [23]. The messages were categorized by the university counselors and researchers from two perspectives: the message content types which included 20 emotional and 18 cognitive messages, aiming to contact students’ deeper emotional and cognitive functions; and the presentation modes which included 17 graphic with directive short messages, seven graphics with motivational short messages, and 14 graphics or motion pictures with long messages, focusing on the tone and structure of how the messages were presented. Figure 1 is a graphic illustration of the emotional type with a directive short message.

The class secretaries would receive the messages from researchers and then post them on the class Facebook group two to four times a week for the duration of 12 weeks (the research period). The secretaries then would collect and return the individual responses (including the number of Likes, Comments, and Likes on these comments) back to the researchers. Since the class teachers and researchers were not members of students’ group, students would not feel restrained while interacting with others in the group. Since the focus of this study is on students’ positive development, only the positive messages were collected.

This study defines two variables to observe the student behaviors: Individual Engagement (IndE), representing the number of Likes and Comments with respect of each message, and Social Influence (SocI), representing the number of Likes on other members’ comments to each message. Moreover, an acceptance test, based on Technology Acceptance Model [23], was conducted at the end of this 12-week event. This acceptance test included three constructs—Perceived Usefulness (PU), Perceived Ease of Use (PEOU), and Intention to Use—that explains the causal relationships related to users’ perception toward the technology in interest. A questionnaire consisting of nineteen 6-point Likert scale questions and three open questions was developed to estimate the acceptance level of this formal event by new college entrants. The open questions were used to assess the benefit and the potential improvements of this event.

### 2.3. Data Collection and Analysis

Data were collected in three phases including an initial survey at the beginning, responses to the posted messages in the middle, and an acceptance test at the end of this experiment. The initial survey will be used to depict subjects’ demographic profile, Internet time and Facebook experiences. For message responses data, four data were collected for each posted message:

- Social Influence (SocI): the numbers of Likes on other posters’ comments to each message.
- Individual Engagement (IndE): the numbers of Likes and Comments on each message. This metric has three levels: 1 = above 41; 2 = between 32 and 41; 3 = below 32.
- Message content type (cType): 1 = emotional, 2 = cognitive.
- Message presentation mode (pMode): 1 = graphic with directive short messages; 2 = graphic with motivational short messages; 3 = graphic or motion picture with long messages.

SPSS Statistics 17.0 (IBM Corporation, Somers, NY, USA) was used for statistical analyses. For the acceptance test, a Bivariate Correlations procedure was used to compute Pearson’s correlation.

## 3. Results and Discussion

### 3.1. Results

#### 3.1.1. Initial Survey

Out of 150 students from two selected classes, 120 (80%) participated in this experiment. Out of these 120 students, 79 (65.8%) were from the medical school and 41 (34.2%) were from the management school; the participants’ age ranged from 18 to 27 years old. The gender distribution was 49 (40.8%) females and 71 (59.2%) males. Their average Facebook experience was 21.4 months, and on the average they spent 30 hours on the Internet and 16.05 hours on Facebook each week. 

#### 3.1.2. Message Responses

The responses collected for 38 positive messages within 12 weeks indicated that for message content types, there were 1,220 Likes, 156 Comments, and 257 Likes on comments. For message presentation modes, there were 566, 219, and 435 Likes for presentation modes 1, 2, and 3, respectively. Table 1 and Table 2 depict the total responses by content type and by presentation mode, respectively. A 3-week interval analysis indicated the behavior differences between the medical and management students, and Table 3 describes the differences between their responses. To evaluate the treatment effect, one-way ANOVA was performed under variant setup. Table 4 is the summary.

**Table 1:** Responses of messages by content type.

cType	N	Likes	Comments	Likes on Comments
emotional	20	661 (352/309)	92 (40/52)	165 (107/58)
cognitive	18	559 (308/251)	64 (45/19)	92 (67/25)
Total	38	1,220 (660/560)	156 (85/71)	257 (174/83)

Note: Numbers are presented as: Subtotal (Med School/Mgmt School).

**Table 2:** Responses of messages by presentation mode.

pMode	N	Likes	Comments	Likes on Comments
1	17	566 (295/271)	88 (39/49)	146 (98/48)
2	7	219 (123/96)	14 (14/0)	37 (37/0)
3	14	435 (242/193)	54 (32/22)	74 (39/35)
Total	38	1,220 (660/560)	156 (85/71)	257 (174/83)

Note: pMode types: 1 = graphic with directive short messages; 2 = graphic with motivational short messages; 3 = graphic or motion picture with long messages. Numbers are presented as: Subtotal (Med School/Mgmt School)

**Table 3:** The average engagements and influence by 3-week interval.

Interval (week)	Likes		Comments		Likes on Comments	
	Medical school	Management school	Medical school	Management school	Medical school	Management school
1–12	17.37	14.61	2.24	1.87	4.58	2.18
1–3	18.09	17.90	2.91	4.12	6.55	3.91
4–6	16.22	14.78	1.56	1.77	5.56	3.67
7–9	19.00	12.38	2.13	1.25	2.38	0.88
10–12	16.30	12.60	2.20	0.00	3.30	0.00

**Table 4:** The treatment ANOVA summary.

Variable	Medical School						Management School					
	Likes		Comments		Likes on Comments		Likes		Comments		Likes on Comments	
	F	P	F	P	F	P	F	P	F	P	F	P
Interval	0.383	0.766	1.185	0.330	1.175	0.333	5.632	0.003 **	2.167	0.100 *	1.717	0.181
cType	0.063	0.803	0.421	0.520	0.486	0.490	1.130	0.294	1.012	0.321	0.613	0.438
pMode	0.005	0.994	0.041	0.959	0.699	0.503	1.182	0.318	0.964	0.391	0.584	0.562

Note: * Significant at 0.1 level; ** Significant at 0.01 level.

Data on Social Influence (SocI) showed a Poisson distribution and thus failed to meet the assumption of normality. The data were subject to a log-normal transformation prior to analysis, and a log-linear model was used to estimate the effect of social influence. The results yielded the following model:

ℓ(SocI) = 1.224 + 1.416I_IndE__ =__ 1_ + 0.981I_IndE__ =__ 2_ + 0.584I_cType__ =__ 1_ + 0.68I_pMode__ =__ 1_ − 0.693I_pMode__ =__ 2_ (1)

The regression model indicates a linear relationship between natural log of social influence and message’s attributes including responses from students, message types, and message presentation modes. This relationship implies that when we control message type 2 (cognitive message) and presentation model 3 (graphic or motion picture with long message) then we can estimate the social influence according to the three levels of individual engagement (IndE = 1, 2, and 3) as 14.00, 9.07, and 3.40, respectively. 

#### 3.1.3. Acceptance Test

**Figure 2:**
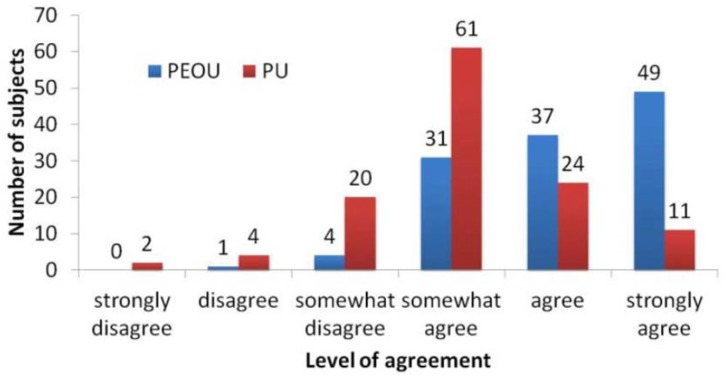
The level of agreements. (PEOU = Perceived Ease Of Use; PU = Perceived Usefulness).

The data analyses from questionnaires indicated that, with respect to the positive messages received, the majority of the students perceived the usefulness and ease of use. Figure 2 shows the level of agreement toward statements in the questionnaire in terms of PU and PEOU constructs. Means and standard deviations for PEOU and PU were 5.05/0.93 and 5.43/0.85, respectively. PEOU was positively correlated with both PU (r = 0.413, *p* < 0.01) and Intention to Use (r = 0.227, *p* < 0.05). PU was positively correlated with Intention to Use (r = 0.675, *p* < 0.01). Table 5 shows the scores of means and standard deviations of each construct as well as the correlations among the constructs. For the open questions at the end of questionnaire, 69 out of 120 students did not respond. Thirty-nine students expressed their thoughts in relation to the effectiveness of the messages, 11 students described the interactions among their classmates, and one student believed that the straight and formal messages have limited effect.

**Table 5:** Correlation matrix.

Construct	Mean	SD	Range	PEOU	PU	Intension to Use
PEOU	5.05	0.93	2 to 6	1.000		
PU	5.48	0.85	1 to 6	0.413 **	1.000	
Intention to use	5.37	0.94	1 to 6	0.227 *	.675 **	1.000

Note: * Significant at 0.05 level; ** Significant at 0.01 level; (1 = strongly disagree; 2 = disagree; 3 = somewhat disagree; 4 = somewhat agree; 5 = agree; 6 = strongly agree).

### 3.2. Discussion

For students in this study at least, Facebook seems to have dominated their Internet time, and this online social network has become a portal for information sharing, activity organizing, and friendship maintenance. Students show no difficulty on accepting this technology, but there are a lot of online events in the network trying to attract their attention. The effectiveness of these events depends on the length of individual engagement and the spread of social influence, thus the metrics of participants’ behavior from these perspectives are crucial for designing online initiatives. The positive messages designed in this study included two content types and three presentation modes were intended to attract student’s attention and motivate them to spread their influences within the social groups. 

#### 3.2.1. Message Responses

From the individual engagement perspective, Table 1 shows that emotional messages, compared to cognitive ones, on the average received slightly more Likes and Comments, and received a lot more Likes on comments. The results of message presentation mode in Table 2 depict that directive short messages, on the average, received slightly more Likes than motivational short messages and long messages, and received a lot more Comments and Likes on comments. Comparing responses from the medical and management school students, medical students commented more on the cognitive messages. The management students commented more on the emotional ones and these received much more Likes on comments in total. Moreover, Table 3 indicates a decreasing tendency in engagement; specifically, while the medical students maintained steady engagement until mid-term and final exams, the management students showed a sharp decrease. Table 4 shows the different reactions between medical and management school students. The management school students preferred emotional messages than cognitive ones (Likes, significant at 0.001 level; Comments, significant at 0.01 level). Overall, the medical school students did not show much preference. However, though not significant, it hinted that students’ reaction to the presentation mode was more obvious than the content type. With respect to the interval, the final examination may have caused the number of responses from the management school students to decrease. The results from above responses may partially support the preference and behavior of this group of students in total and difference between two schools on engaging online message types and message presentation modes, as found in [24,25]. 

From a social influence perspective, the results from the regression model indicated that for this group of students, using certain types of positive messages with various presentation modes can estimate social influence under certain numbers of individual engagements to a message. For instance, if we choose to post a message with type 1 (emotional message) and presentation model 3 (graphic or motion picture with long message) then we can estimate social influence according to three levels of individual engagement (IndE = 1, 2, and 3) as 25.12, 16.26, and 12.30 respectively. In other words, if a practitioner designed a Facebook event then she can choose to post a message in the event on certain type and mode and estimate social influence by different levels of individual engagement. Measuring Facebook activities especially on engagement and influence issues has attracted many attentions and debates. It is difficult to force individuals to engage in such free environment -- for instance, submit Like and Comment; however, individuals in social groups may be influenced by other members’ engagements and submit Like on these members’ comments. The number of engagements may partially depend on individual’s preference, but the social groups where individuals participating in can have certain influence to them and to the success of an online event [10]. This study established the relationship between individual engagement and social influence with a Log-linear model that can partially explain the activities and behavior of the new entrants and be used for future researches. 

#### 3.2.2. Acceptance Test

The results from acceptance evaluation indicated that this intervention is perceived usefulness and ease of use and most of the students appreciated the positive messages posted on their Facebook groups. However, over half of the students did not respond to the open question implying that a formal information collecting method requiring students to express their private opinions may not suitable in this situation. For those who did respond, many students believed this event can motivate their positive thinking and improve understanding of their classmates. They also mentioned being cautious about message selection that appeared too formal or improper messages that may cause negative effects for the whole event.

#### 3.2.3. Limitation

This preliminary study about positive psychology development and measurement of online social network application is far from a theoretical-sounded approach in terms of sample size and definitions of individual engagement and social influence. However, this study does try to measure social influence with individual engagement and a designed positive message promotion event on a group of new college entrants. The findings can partially explain the behavior of the students toward this online event and advance our understanding on designing positive development events on students’ online social networks.

## 4. Conclusions

Promoting positive traits can have substantial impact on adolescents’ future development and many of positive traits can be cultivated partially by emotional events and social influence. Based on a positive psychology framework, this study designed a positive message delivery approach to motivate positive development of a group of new university entrants through their Facebook groups. This study collected students’ responses and measured the relationship between individual engagement and social influence that contributes to the understanding of dynamics in the Facebook activities and provides references for designing online social network events. 

A benefit of using class-based groups in Facebook is that this can promote a positive atmosphere that enhances strong relationships ties both in the physical and virtual environments for new college entrants. When they pass the freshman period, the class-based strong ties relationships may transfer to weak ties that may have a chance to broaden the influence of positive message practices. As online social networks become major tools for college students, how to study such influence on students’ daily activities and develop proper positive trait development methods is crucial in this trend.
